# Analysis of HIF2α polymorphisms in infertile women with polycystic ovary syndrome or unexplained infertility

**DOI:** 10.3389/fendo.2022.986567

**Published:** 2022-09-08

**Authors:** Xiaoya Zheng, Jiani Ma, Min Hu, Jian Long, Qiang Wei, Wei Ren

**Affiliations:** ^1^ Department of Endocrinology, the First Affiliated Hospital of Chongqing Medical University, Chongqing, China; ^2^ Reproductive and Infertility Center, the First Affiliated Hospital of Chongqing Medical University, Chongqing, China; ^3^ Prevention of Disease Department, Chongqing Jiulongpo District Hospital of Traditional Chinese Medicine, Chongqing, China

**Keywords:** infertility, HIF2α gene, polymorphism, obesity, insulin

## Abstract

**Objective:**

To evaluate HIF2α polymorphisms and glucose metabolism in a group of women with polycystic ovary syndrome (PCOS) or unexplained infertility (UI).

**Patients:**

The infertile group (n=148) consisted of 96 women with PCOS, 52 women with UI, and176 women without infertility as a healthy control group.

**Intervention:**

We genotyped 29 single nucleotide polymorphisms (SNPs) of HIF2α by using matrix-assisted laser desorption/ionization time-of-flight mass spectrometry (MALDI-TOF MS)-based genotyping technology. The genetic associations were analyzed statistically.

**Main outcome measures:**

Allele frequency, genotype distribution and haplotype analyze of the HIF2α polymorphisms were performed. Body mass index (BMI), waist circumference, uric acid (UA), high-sensitivity C-reactive protein (hsCRP), lipids, glucose and insulin tolerance - were also measured.

**Results:**

Infertile women with PCOS had a higherBMI and waist circumference, elevated hsCRP and uric acid (UA) levels, impaired glucose tolerance, and increased levels of plasma insulin compared to UI patients and healthy women. SNP analysis of HIF2α revealed that the allele and genotype frequencies of rs4953361 were significantly associated with infertile women with PCOS. Haplotype analysis of the HIF2α polymorphism identified haplotypes TGG and TGA as being associated with infertile women with PCOS. Women with the AA genotype of rs4953361 had a significantly higher BMI and post load plasma glucose and insulin levels than those of women with the GG genotype.

**Conclusion:**

Infertile women with PCOS more commonly have metabolic disturbances than those with UI. This is the first study to report an association between HIF2α polymorphisms (rs4953361) and the risk of infertile women with PCOS, not UI, in Han Chinese population. These results require replication in larger populations.

In this observational study, we did not report the results of a health care intervention on human participants. The study was approved by the Human Research Ethics Committee of the First Affiliated Hospital of Chongqing Medical University. Clinical data and peripheral blood samples were collected only after explaining the objectives of the study and obtaining a signed informed consent form.

## Introduction

Infertility is a problem in both developed and developing countries worldwide (1). Currently, 15% of the world’s population suffers from infertility, and half of them are women (2). Several factors, such as aging, neuroendocrine, infectious, immune, psychiatric, stress, and weight factors, as well as iatrogenic factors, previous interventions, and surgery, have been claimed to affect women’s fertility (3). In addition, several studies have revealed many genetic factors related to female infertility that indicate associations between the development of female infertility and genetic polymorphisms (4, 5). Polycystic ovary syndrome (PCOS) is a common disorder with wide-ranging clinical heterogeneity that causes infertility, insulin resistance and impaired glucose metabolism. However, the comprehensive molecular mechanisms of PCOS in causing infertility remain unclear (6). Unexplained infertility (UI) means the inability of couples to become pregnant after a year without obvious male or female infertility factors. Unfortunately, nearly one-third of infertility patients are unaware of the cause (7). Due to the lack of understanding of the pathogenesis, the treatment of UI has been a complex challenge in the field of reproductive medicine (8).

Hypoxia, a status caused by reduced oxygen availability or an imbalance in oxygen consumption/supply, is a stress that affects many physiological and pathological processes (9). Hypoxia inducible factor 2α (HIF2α) is a major transcription factor that responds to hypoxia and induces the expression of hypoxia-related genes such as vascular endothelial growth factor (VEGF) and erythropoietin (10). Notably, HIF2α is strongly expressed in the uterine stroma after embryo attachment (11). Based on the expression patterns, a study reported that aberrant expression of HIF2α in the entire uterus of mice resulted in infertility, indicating the importance of uterine HIF2α in infertility (12). A study reported that the disrupted hypoxic signaling pathway in the granulosa-lutein cells of periovulatory follicles in PCOS women might play a role in ovulation failure and in the reduction in fertility prevalent in this syndrome. Another study reported that the failure of transformation to glycolysis and low HIF1α expression in granulosa cells during the development of follicles might be correlated with the low oocyte competence of PCOS (13). Wang et al. found that the HIF1α signaling pathway might be an important mechanism regulating PCOS formation and treatment in mammalian ovaries *in vivo* and should be a new clinical target for PCOS prevention and treatment in the future (14). To date, whether HIF2α polymorphisms are associated with PCOS and UI has not been reported.

As there is emerging evidence on the potential contribution of HIF2α in modulating female infertility, the current study aimed to explore the relationship between genetic polymorphisms of HIF2α and female infertility with PCOS or UI.

## Research design and methods

### Participants

In this case–control study, women with infertility who visited the reproductive center of our hospital from January 2020 to December 2021 were screened. Women were included if they had one or more years of infertility, and the male partner was screened to rule out for conditions that cause infertility. The patients underwent detailed clinical assessment and determination of the causes of infertility. Tubal occlusion and endometriosis patients were excluded. Patients with uterine fibroids or malformation were also excluded. Patients with hypothalamic-pituitary-adrenal diseases or known diabetes were also excluded. Finally, 96 women with PCOS diagnosed according to the Rotterdam criteria (15) and 52 women with UI were enrolled as the case group. A total of 176 age-matched healthy women not seeking fertility treatment and without any known fertility problems who underwent routine health check-ups during the same period were enrolled as the control group. Subjects in the control group were a general public cohort with regular menses at 22- to 35-day intervals, and they did not have a history of infertility. Women with any known acute or chronic heart, liver, renal, autoimmune or endocrine diseases or known or suspected tumors were excluded. Clinical data and peripheral blood samples were collected only after explaining the objectives of the study and obtaining a signed informed consent form, as approved by the Human Research Ethics Committee of our hospital.

### Anthropometric measurements and laboratory tests

Anthropometric measurements included height, weight, waist circumference and blood pressure. BMI was calculated as weight (kg) divided by the square of height (m). Blood biochemical analyses included cholesterol (TC), triglycerides (TG), low-density lipoprotein cholesterol (LDL-c), high-density lipoprotein cholesterol (HDL-c), uric acid (UA), high-sensitivity C-reactive protein (hsCRP), and glycosylated hemoglobin (HbA1c). These indicators were measured by an immuno-chemical-automated analyzer (Type 7600, Hitachi Ltd., Japan). A 75-g oral glucose tolerance test (OGTT) and insulin release test (IRT) were performed in the endocrinology laboratory of our hospital as previously described (16). Blood plasma glucose was measured using the glucose oxidase method. Blood plasma insulin was measured by the chemiluminescence method.

### DNA extraction

Peripheral blood was collected from each patient and control in an ethylene diamine tetra acetic acid (EDTA)-containing tube. Genomic DNA was extracted from peripheral nucleated cells by using a commercially available kit (Axygen, JA1705).

### HIF2α genotyping

For detection of HIF2α polymorphisms, 29 tag SNPs of HIF2α were genotyped by using matrix-assisted laser desorption/ionization mass spectrometry (MALDI-TOF MS) as previously described (17). Genotyping was performed using MALDI-TOF MS (MassArray™ Nanodispenser, SAMSUNG). All reactions were designed in multiplexes of up to 29 tag SNPs usingAssay Design v2.0 software (Agena Bioscience). The data were collected using the Mass ARRAY Compact System (Agena Bioscience). Detailed primer information is provided in **Supplementary Material 1**.

### Statistical analysis

Statistical analysis was performed by GraphPad Prism version 5.0 software. Continuous variables with a normal distribution are expressed as the mean ± standard deviation (mean± SD). Multiple-group comparisons of means were performed using two-way analysis of variance followed by two group comparison. Categorical variables are described as percentages (%), and all SNPs were tested for Hardy–Weinberg equilibrium with the chi-square test. Haploview software developed at the Massachusetts Institute of Technology (MIT) Media Lab by B. Fry (http://acg.media.mit.edu/people/fry/) was used to perform the haplotype analysis (18). All statistical analyses were two-sided, and p<0.05 was considered statistically significant.

## Results

**Figure 1:**
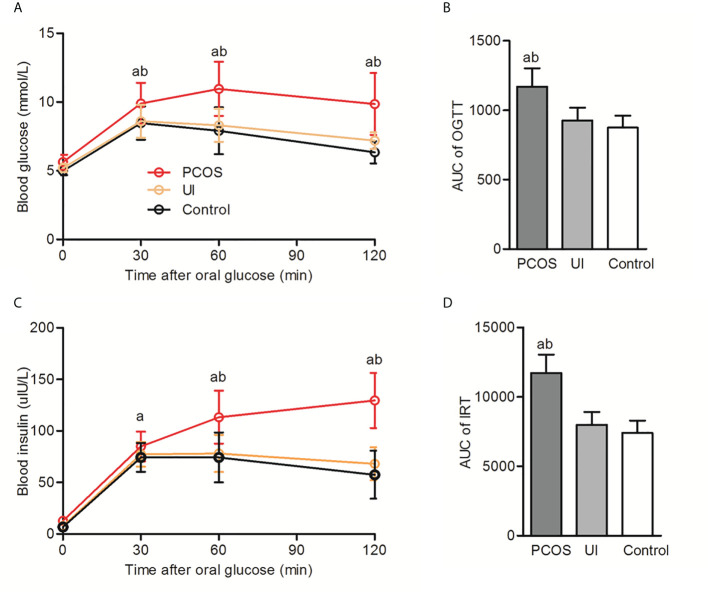
Oral glucose tolerance test (OGTT) and insulin release test (IRT) in infertile patients with and without PCOS and in the control group. **(A, B)**: Comparison of the oral glucose tolerance test (OGTT) and corresponding area under the curve (AUC) among PCOS, UI and the control group. **(C, D)**: Comparison of insulin release test (IRT) and corresponding area under the curve (AUC) among PCOS, UI and the control group. Two-way ANONA followed by two group comparison. ^a^P < 0.05 compared with control group, ^b^P < 0.05 compared with UI group.

Comparative analysis of the clinical characteristics of the studied population is shown in **Table 1**. Infertile women with PCOS had a significantly higher BMI and waist circumference than UI and healthy women (p<0.05). Infertile women with PCOS also had significantly higher hs-CRP and UA levels than UI and healthy women. There were no significant differences in blood pressure or blood lipids between the infertile and control groups. We found no significant differences in fasting blood glucose among those groups, but blood glucose and insulin levels in infertile patients with PCOS were significantly higher than UI and healthy women after a glucose tolerance test (**Figure 1**).

**Table 1:** Clinical characteristics of the studied population.

Characteristics	Infertility (n = 148)		Control (n = 176)
	PCOS(n = 96)	UI (n = 52)	
Age (years)	29.28 ± 4.40	29.32 ± 4.20	29.10 ± 3.91
BMI (Kg/m^2^)	26.80 ± 3.72^ab^	21.6 ± 3.51	20.92 ± 3.22
Waist (cm)	88.30 ± 9.91^ab^	79.82 ± 9.64	76.24 ± 6.61
SBP (mmHg)	117.2 ± 17.5	108.5 ± 18.2	112.4 ± 10.2
DBP (mmHg)	75.8 ± 13.7	72.6 ± 14.3	71.8 ± 9.1
TC (mmol/L)	4.84 ± 1.26	3.62 ± 1.41	3.28 ± 0.86
TG (mmol/L)	1.68 ± 0.82	0.84 ± 0.72	0.79 ± 0.44
HDL-c (mmol/L)	0.92 ± 0.32	1.12 ± 0.32	1.21 ± 0.54
LDL-c (mmol/L)	2.66 ± 0.93	1.56 ± 0.83	1.48 ± 0.32
HbA1c (%)	5.62 ± 1.64	4.92 ± 1.21	4.86 ± 1.14
UA (μmol/L)	272 ± 48.1^ab^	274.3 ± 52.2	238.8 ± 30.8
HsCRP (mg/L)	8.66 ± 4.83^ab^	4.22 ± 3.21	3.16 ± 2.42

BMI, body mass index; DBP, diastolic blood pressure; HbA1c, glycated hemoglobin; HDL-c, high density lipoprotein-cholesterol; LDL-c, low-density lipoprotein-cholesterol; PCOS, polycystic ovary syndrome; SBP, systolic blood pressure; TC, total cholesterol; TG, triacylglycerol; UA, uric acid; UI, unexplained infertility.

a P < 0.05, compared with the control group.

b P < 0.05, compared with the UI group,the difference was statistically significant.

For 29 tag SNPs of HIF2α, except for rs11694193, the observed and expected values of alleles and genotypes were in good agreement with Hardy–Weinberg equilibrium (p>0.05). The allele and genotype frequencies of SNPs between the infertility and control groups that were significantly different (p<0.05) and close to significantly different (p<0.1) are shown.

The allele frequencies of the HIF2α polymorphisms rs2346176, rs4953361, and rs13412887 in infertile women with PCOS or UI and healthy women are shown in **Table 2**. We found a significant association between the rs4953361 polymorphism and infertile patients with PCOS (P=0.005, OR= 1.65, 95% CI 1.16–2.35), infertile women with PCOS had significantly higher frequencies of A allele than healthy women. These results suggested that the rs4953361 polymorphism was related to infertile women with PCOS.

**Table 2:** Allele frequencies of the HIF2α polymorphisms in infertile patients with PCOS or UI and in the control group.

HIF2α polymorphisms	Population studied	Alelles n (%)		OR^a^(95%CI)^a^ *P*-value^a^	OR^b^(95%CI)^b^ *P*-value^b^	OR^c^(95%CI)^c^ *P*-value^c^
		C	T			
rs2346176	PCOS	139 (0.72)	53 (0.28)	1.48	1.20	1.30
	UI	78 (0.75)	26 (0.25)	(0.99-2.23)	(0.70-2.01)	(0.77-2.17)
	Controls	280 (0.80)	72 (0.20)	0.06	0.50	0.32
		G	A			
**rs4953361**	PCOS	82 (0.43)	110 (0.57)	**1.65**	1.51	1.09
	UI	55 (0.53)	49 (0.47)	**(1.16-2.35)**	(0.93-2.43)	(0.71-1.70)
	Controls	194 (0.55)	158 (0.45)	**0.005**	0.09	0.69
		G	C			
rs13412887	PCOS	39 (0.20)	153 (0.80)	1.50	1.22	1.24
	UI	18 (0.17)	86 (0.83)	(0.95-2.38)	(0.66-2.26)	(0.69-2.23)
	Controls	51 (0.14)	301 (0.86)	0.08	0.53	0.48

OR, odds ratio; CI, confidence interval; PCOS, polycystic ovary syndrome; UI, unexplained infertility; P < 0.05 indicates statistical significance and is shown in bold. For rs4953361 of HIF2α, patients with PCOS had significantly higher frequencies of A allele and lower G allele than UI and control subjects (p = 0.005).

a PCOS vs control.

b PCOS vs UI.

c UI vs control.

The genotype frequencies of the HIF2α polymorphisms rs2346176, rs4953361, and rs13412887 in the women with and without infertility are shown in **Table 3**. The genotype frequency distribution of rs4953361 between the infertile women with PCOS and healthy women was significantly different (p=0.02). The frequency of the AA genotype of rs4953361 in infertile women with PCOS was significantly higher than healthy women (32% vs. 21%, p=0.02), indicating that women with AA genotype of rs4953361 had a higher risk of infertility due to PCOS.

**Table 3:** Genotype frequency distribution of the HIF2α polymorphisms in infertile patients with PCOS or UI and in the control group.

HIF2αpolymorphisms	Population studied	Genotypes n (%)			*P*-value^a^	*P*-value^b^	*P*-value^c^
		CC	CT	TT			
rs2346176	PCOS	52 (0.52)	35 (0.39)	9 (0.09)			
	UI	30 (0.58)	18 (0.35)	4 (0.07)			
	Controls	111 (0.63)	58 (0.33)	7 (0.04)	0.09	0.39	0.23
		AA	AG	GG			
**rs4953361**	PCOS	31 (0.32)	48 (0.50)	17 (0.18)			
	UI	12 (0.23)	25 (0.48)	15 (0.29)			
	Controls	37 (0.21)	84 (0.48)	55 (0.31)	**0.02**	0.23	0.93
		CC	CG	GG			
rs13412887	PCOS	62 (0.65)	29 (0.30)	5 (0.05)			
	UI	36 (0.69)	14 (0.27)	2 (0.04)			
	Controls	127 (0.72)	47 (0.27)	2 (0.01)	0.09	0.83	0.42

PCOS, polycystic ovary syndrome; UI, unexplained infertility; P < 0.05 indicates statistical significance and is shown in bold. For rs4953361 of HIF2α, patients with PCOS had significantly higher frenquncies of AA genotype and lower GG genotype than UI and control subjects (p = 0.02).

a PCOS vs control.

b PCOS vs UI.

c UI vs control.

**Figure 2:**
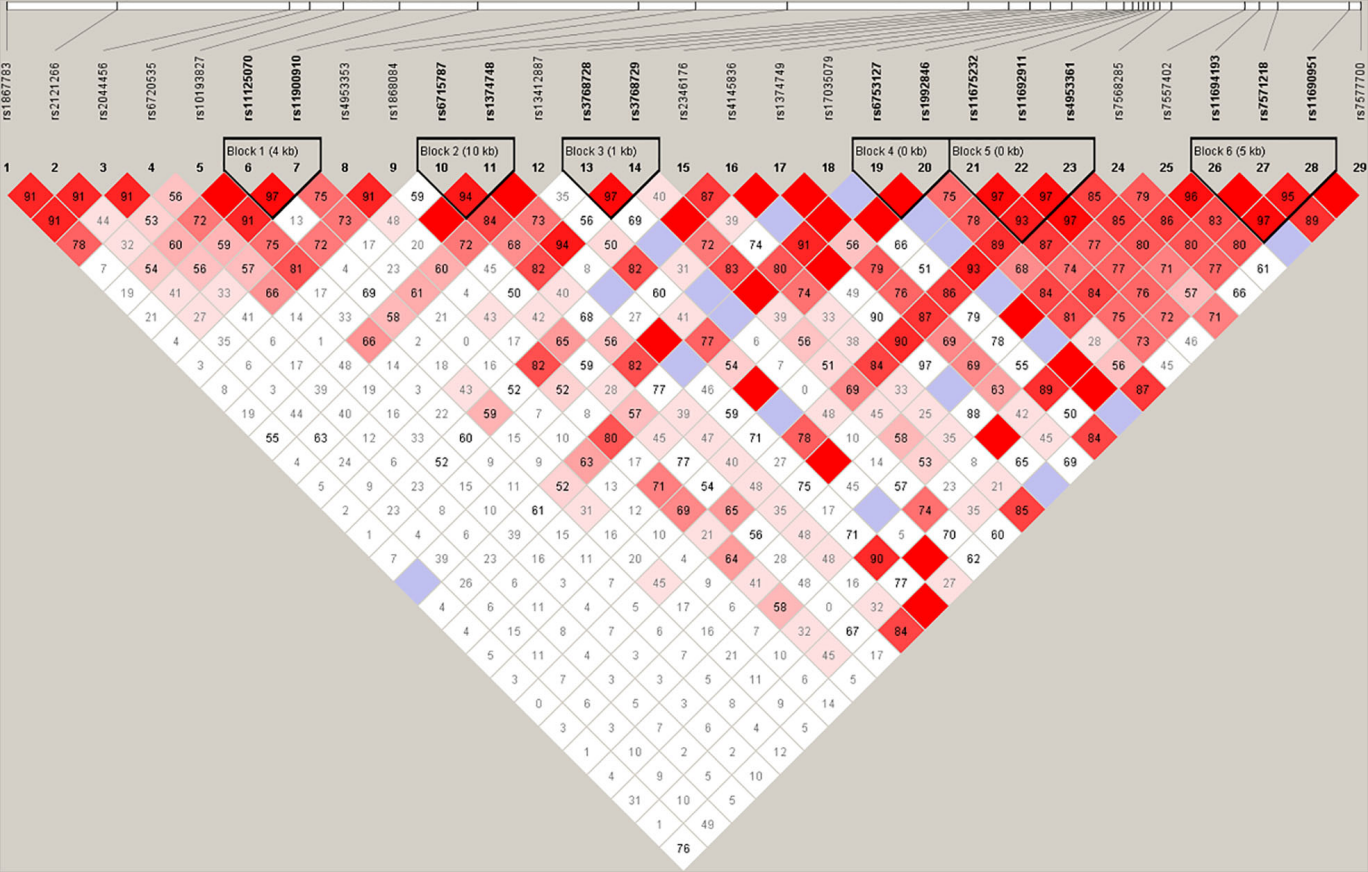
Haplotype analysis. Haplotype analysis showed that rs11675232, rs11692911, and rs4953361 of HIF2α constituted block 5, and the distributions of haplotypes TGG and TGA in infertile women with PCOS and healthy women were significantly different (p < 0.05).

**Figure 3:**
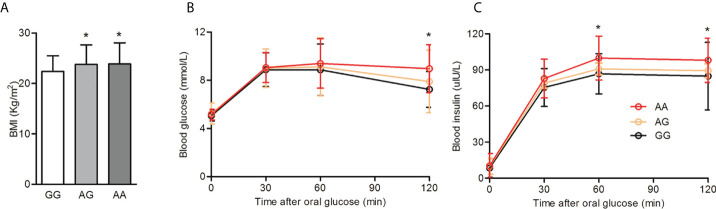
BMI and glucose tolerance among different genotypes of rs4953361. **(A)**: Comparison of BMI among different genotypes of rs4953361. **(B, C)**: Comparison of the OGTT and IRT among different genotypes of rs4953361. *p < 0.05 compared with the GG genotype.

Haplotype analysis of the HIF2α polymorphisms identified that rs11675232, rs11692911, and rs4953361 constituted block 5 (**Figure 2** and **Table 4**). The haplotype frequency of TGG in infertile women with PCOS was significantly lower than those healthy women (42% vs. 54%, p=0.01). In contrast, the haplotype frequency of TGA in infertile women with PCOS was significantly higher than healthy women (37% vs. 26%, p=0.0093). These data suggested that women with TGA haplotype had a higher risk of infertility due to PCOS. In the subgroup analysis of the different genotypes at rs4953361, we found that women with the AA and AG genotypes had a significantly higher BMI than women with the GG genotype (23.87 ± 4.16 and 23.77 ± 3.87 vs. 22.37 ± 3.09, P=0.0295). Women with the AA genotype had significantly higher plasma glucose levels at 120 min after OGTT than women with the GG genotype. Women with the AA genotype had significantly higher plasma insulin levels at 60 min and 120 min after OGTT than women with the GG genotype (**Figure 3**). These results indicated that women with homozygous or heterozygous mutation of the A allele had a higher risk of increased BMI and impaired glucose and insulin tolerance.

**Table 4:** Haplotype analysis between women with and without infertility.

Block	Haplotypes	Frequencies					
		PCOS	UI	Control	*P*-value^a^	*P*-value^b^	*P*-value^c^
Block 5							
0.67	**TGG**	**0.42**	**0.52**	**0.54**	**0.01**	0.11	0.67
0.77	**TGA**	**0.37**	**0.28**	**0.26**	**0.01**	0.11	0.77
0.90	CAA	0.16	0.17	0.17	0.85	0.80	0.90
0.72	CGA	0.02	0.01	0.01	0.56	0.47	0.72

Rs11675232, rs11692911, and rs4953361 constitute block 5, and haplotype analysis revealed the haplotypes TGG and TGA between the groups of PCOS and control are statistically significant. The P value was 0.01 for haplotype TGG that was associated with a lower risk of PCOS, and the P value was 0.01 for haplotype TGA that was associated with a higher risk of PCOS. P < 0.05 indicates statistical significance and is shown in bold.

a PCOS vs control.

b UI vs control.

c PCOS vs UI.

## Discussion

In the present study, we hypothesized that HIF2α polymorphisms might be associated with infertility caused by PCOS or UI in females. We examined 29 HIF2α SNPs in infertile women with PCOS or UI, and assessed the association of the allele, genotype and haplotype frequencies with the risk of female infertility and glucose metabolism. To our knowledge, this is the first study to report an association between HIF2α polymorphisms and female infertility due to PCOS or UI.

Here, we observed that increased BMI and metabolic disorder and insulin resistance (IR) were characteristics of infertile women with PCOS, but not UI. PCOS can lead to obesity; on the other hand, the increasing obesity epidemic, with its related metabolic disorders, is associated with an increased risk of many female reproductive conditions (19). Reduced fertility in women who are overweight might be related to multiple endocrine, adipokine, and metabolic alterations that affect follicle growth, embryo development and implantation (20). Insulin resistance (IR) is another important factor that contributes to the pathophysiology of PCOS associated infertility. Plasma levels of insulin are correlated with BMI, and insulin can regulate steroidogenesis in ovarian cells *in vitro* and in the stromal and follicular compartments of ovaries (21, 22). Xu et al. found elevated plasma insulin levels along with increased glucose and insulin pathway dysfunction in overweight/obese women, these factors have been shown to be associated with reduced follicle-stimulating hormone receptor (FSHR) expression, estrogen synthesis-related genes, and decreased estrogen production (23).

We also found that infertile women with PCOS had significantly higher hs-CRP and UA than healthy women. Hu et al. reviewed UA involvement in female reproductive disorders. High levels of UA can lead to systemic sterile inflammation and may also become a proinflammatory factor. Excessive accumulation of UA might cause oxidative stress, inflammation, metabolic disorders and female reproductive disorders to multiple systems throughout the body (24). Another interesting finding of this study was that although there were no significant differences in fasting blood glucose and HbA1c levels between infertile and healthy women, blood glucose and insulin levels in infertile patients with PCOS were significantly higher than those in UI and healthy women after a glucose tolerance test. This result suggests that it is not enough to check fasting blood glucose and HbA1c levels only for infertile women with PCOS. To avoid missing the diagnosis of patients with impaired glucose tolerance and insulin resistance, it is recommended to perform glucose and insulin tolerance tests.

Regarding the analyzed HIF2α polymorphisms, among the 29 SNPs, the only significant association detected was between rs4953361 and female infertility. Infertile women with PCOS were significantly associated not only with the allele and genotype distribution frequencies of rs4953361, but also the distribution of haplotypes containing this locus. These results suggested that rs4953361 of HIF2α was an important polymorphism site associated with infertility due to PCOS. HIF has been postulated to be involved in follicle development due to the hypoxic microenvironment in follicles (25). However, it has become clear that the expression of HIF1α is regulated by pituitary hormones instead of hypoxia in follicles (26). A previous study showed that the expression of HIF1α is primarily in the uterine luminal epithelium during the peri-implantation period. Interestingly, HIF2α is strongly expressed in the uterine stroma during peri-implantation and after embryo attachment (11). To explore the functional roles of HIF2α in embryo implantation, Matsumoto et al. generated mice with uterine tissue-specific deletion of HIF1α and HIF2α. They found that mice with HIF2α deletion in the entire uterus were infertile, whereas mice with uterine deletion of HIF1α showed subfertility, indicating the importance of HIF2α in fertility. Uterine HIF2α contributes to successful implantation regardless of decidualization and the position of embryo attachment (12). The mechanism of how the rs4953361 polymorphism, an intronic mutation, affects female infertility still needs to be clarified in future studies.

As an additional insight, we compared the BMI, glucose tolerance, and insulin release among women with different genotypes of rs4953361. We found that compared with women with the GG genotype, women with the AA genotype had a significantly higher BMI, two-hour post load plasma glucose level, and one- to two-hour post load plasma insulin levels. These results indicated that the rs4953361 mutation in HIF2α might be associated with obesity and its related impairment in glucose and insulin tolerance. The obesity-associated adipose tissue hypoxia response is largely mediated by HIFs. Adipocyte-specific HIF2α knockout exacerbated high-fat diet-induced inflammation and insulin resistance (27). Adipocyte HIF2α protects against maladaptation to obesity and metabolic dysregulation by promoting angiogenesis in both white adipose tissue and brown adipose tissue and by counteracting obesity-mediated brown adipose tissue dysfunction (28). Macrophage HIF2α has been shown to counteract proinflammatory responses to relieve obesity-induced insulin resistance in adipose tissue (29, 30). Taken together, these studies indicate that HIF2α is an important regulator of obesity and insulin resistance. In our study, we found that the rs4953361 polymorphism of HIF2a may be associated with elevated BMI and metabolic disorder, especially impaired glucose tolerance and insulin release in infertile women with PCOS, but not UI.

There are some limitations of this study that should be mentioned. First, we may not have enough power to detect a potential association between the other 27 tag SNPs of HIF2α and female infertility due to the limited sample size, or differences in genetic effect sizes. Second, healthy women lack sex hormone levels at baseline, making it impossible to compare sex hormone differences between groups. Third, the population included in this study is a single center in western China, and the association between the rs4953361 polymorphism of HIF2α and female infertility and metabolic disorders still needs to be validated in other populations.

## Conclusion

In this study, we found that infertile women with PCOS more commonly had metabolic disturbances than those with UI. Our data point to a possible association of the HIF2α polymorphism (rs4953361) with women infertility due to PCOS in Han Chinese population. This finding clearly needs to be replicated in a larger and independent sample and in different populations. The mechanism of the effect of the HIF2α polymorphism rs4953361 on female infertility still needs to be clarified in future studies.

## Data availability statement

The original contributions presented in the study are included in the article/**Supplementary Material**. Further inquiries can be directed to the corresponding author.

## Ethics statement

The studies involving human participants were reviewed and approved by the study was approved by the Human Research Ethics Committee of the First Affiliated Hospital of Chongqing Medical University. The patients/participants provided their written informed consent to participate in this study.

## Author contributions

XZ designed and wrote the manuscript. JM and MH collected the data. JL and QW analyzed the data. WR is the design consultant. All authors contributed to the article and approved the submitted version.

## Funding

This project was supported by the National Natural Science Foundation of China Youth Program (Grant No.81900733) in design of the study, the Chongqing municipal health and Family Planning Commission Fund (Number: 2021MSXM013) in collection of data, Bethune Charity Foundation (G-X-2020-1107-14) in interpretation of data, and the National Key Clinical Specialties Construction Program of China (2011) in writing and publication the manuscript.

## Conflict of interest

The authors declare that the research was conducted in the absence of any commercial or financial relationships that could be construed as a potential conflict of interest.

## Publisher’s note

All claims expressed in this article are solely those of the authors and do not necessarily represent those of their affiliated organizations, or those of the publisher, the editors and the reviewers. Any product that may be evaluated in this article, or claim that may be made by its manufacturer, is not guaranteed or endorsed by the publisher.
